# Brief Report From the 3rd International Meeting on Bone Marrow Adiposity (BMA 2017)

**DOI:** 10.3389/fendo.2019.00336

**Published:** 2019-05-28

**Authors:** Alessandro Corsi, Biagio Palmisano, Josefine Tratwal, Mara Riminucci, Olaia Naveiras

**Affiliations:** ^1^Department of Molecular Medicine, Sapienza University of Rome, Rome, Italy; ^2^Institute of Bioengineering (IBI) and Swiss Institute for Experimental Cancer Research (ISREC), École Polytechnique Fédérale de Lausanne (EPFL), Lausanne, Switzerland; ^3^Hematology Service, Departments of Oncology and Laboratory Medicine, Centre Hospitalier Universitaire Vaudois (CHUV), Lausanne, Switzerland

**Keywords:** bone marrow adiposity, bone marrow adipocytes, bone marrow, bone marrow adiposity society (BMAS), bone, bone marrow fat, bone metabolism, yellow marrow

## Abstract

The 3rd International Meeting on Bone Marrow Adiposity (BMA) was held at the Olympic Museum in Lausanne, Switzerland, on August 31st and September 1st, 2017. This brief monograph summarizes the scientific contents of the meeting and highlights the birth of the International Bone Marrow Adiposity Society (BMAS).

## Introduction

A two-day meeting on Bone Marrow Adiposity (BMA) was held at the Olympic Museum in Lausanne, Switzerland, on August 31st and September 1st, 2017 (https://bma2017.sciencesconf.org). The meeting was co-organized by Olaia Naveiras, Alessandro Corsi, and Mara Riminucci with the active collaboration of Biagio Palmisano and Josefine Tratwal, as a joint effort between their laboratories at the Ecole Polytechnique Federale de Lausanne (EPFL) and the Sapienza University of Rome. This meeting was dedicated to the memory of the excellent scientist and expert in bone and bone marrow (BM) physiopathology, Paolo Bianco, anatomical pathologist at the Sapienza University of Rome who passed away in 2015 (1, 2).

In continuity with the first two meetings held in Lille, France [https://bma2015.sciencesconf.org; (3)], and in Rotterdam, the Netherlands [https://adiposity.sciencesconf.org/; (4)], the 3rd International Meeting on BMA was organized to provide physicians and scientists worldwide with different backgrounds -including bone biology, metabolism, oncology, endocrinology, hematology, and rheumatology- the opportunity to share novel results and exchange views on the emerging field of BMA (5–17). Reflecting the focus of the hosting laboratories, emphasis was placed on the bioengineering aspects and stem cell biology perspectives in BMA.

**Figure 1:**
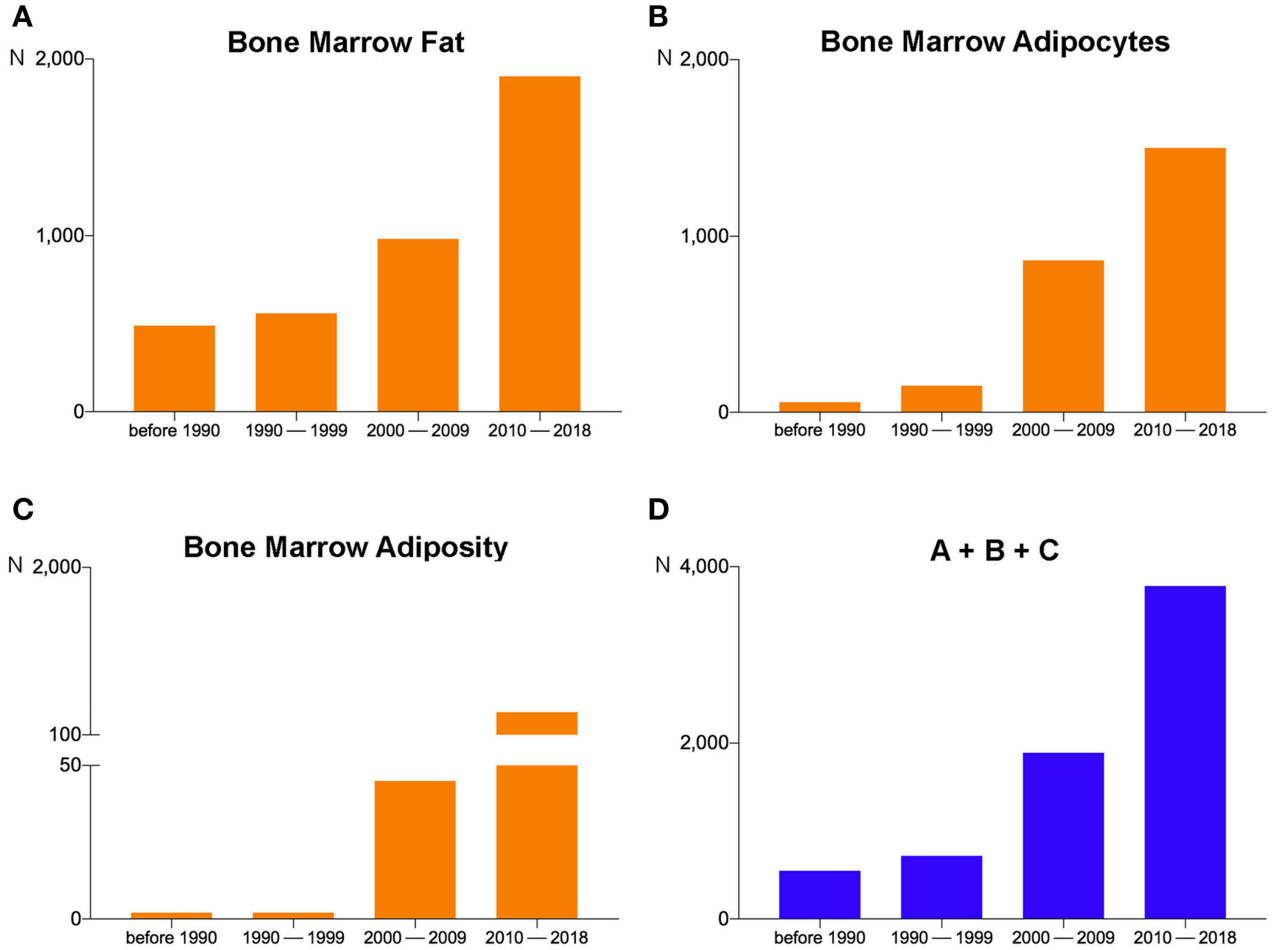
Pubmed search results on September 10, 2018 for **(A)** “Bone Marrow Fat,” **(B)** “Bone Marrow Adipocytes,” and **(C)** “Bone Marrow Adiposity” revealed 3,929, 2,571, and 428 items, respectively. For each search, the period from 2010–2018 contains the largest number of items. **(D)** The sum of the items obtained by the three different searches (A + B + C). The number of the total items in 2010–2018 has grown by about 100%, 500%, and 700%, respectively, compared to 2000–2009, 1990–1999, and before 1990.

Through recent years, the increasing focus of sessions and symposia on BMA together with the invitation of BMA experts to congresses organized by international societies across different fields (endocrinology, hematology, bone, imaging) underlines the important implications of BMA in metabolic homeostasis, pathologic conditions (osteoporosis, diabetes, obesity, anorexia nervosa, blood diseases, cancer), and aging. Based on this broad and impactful relevance, documented also by the growing number of scientific articles published on this topic in recent years (Figure 1), the need to potentiate basic, translational, and clinical research on BMA was reflected at the constitutive assembly of the BMA meeting in Lausanne, where attendees voted to approve the creation of the International BMA Society (BMAS; http://bma-society.org/) as detailed below.

## Overview of the Scientific Sessions

The meeting focused on varying aspects of BMA, from the developmental origin, functional properties, and endocrine and paracrine regulation of BM adipocytes to the relationship between BM adipocytes in hematopoiesis and with bone tissue. It also included novel technologies and engineering approaches for assessing BMA and the role of BMA in disease. Sixty-six delegates from 14 different countries traveled from as far as China, South Africa, the USA, and across Europe to attend the two-day meeting. The seven invited speakers came from France, Denmark, the USA, and China, bringing fresh aspects of their research fields to the multidisciplinary BMA community.

Twenty-four abstracts were received and ranked by an external committee, resulting in 17 oral presentations and seven poster presentations with a dedicated poster pitching session. Prizes for best oral presentations were awarded to the top scoring abstract, “Regulation of bone marrow adipocytes by the sympathetic nervous system: from bone to brain” by *EL Scheller*, and the top junior presentation, “Biomimetic engineering of a functional *ex vivo* human hematopoietic niche” by *T Klein*. Further echoing the high scientific content of BMA 2017, the contents of many of the oral and poster presentations have been published immediately before or after the meeting (18–35). Even though this long list of published papers unequivocally attests the recent advances in understanding the development, properties and (endocrine and paracrine) regulation of BMA, in clarifying the strong relationship between BMA and haematopoiesis/skeletal tissue and the role of BMA in disease as also the improvement of the technological approaches for assessing BMA, the need for more in-depth research emerged in all scientific sessions. Challenges associated to the lack of uniform nomenclature and lack of standardization of the methodologies specific to BMA and research were recurrent comments during the discussions, reason why the first tasks assigned to the newly created working groups were the preparation of consensus documents in Nomenclature and BMA methodologies, which were discussed in the subsequent meetings among the members of each of the two groups and whose final form accompanies this Frontiers in Endocrinology Bone Research Section issue.

A debate session on the origin of BM adipocytes among three community leaders (*MC Horowitz, T Schulz*, and *BO Zhou*) with an equally prominent moderator (*M Kassem*) was also organized. The heterogeneity of BM adipocytes and the methodological difficulties in tracing their origin were uncovered through the distinct perspectives that emerged during the discussion. A consensus was reached at the meeting that BMA is indeed heterogeneous depending on skeletal location, with regionalization based on genetic tracers pinpointing to specific differences in limbs and tail BM adipocytes as compared to the axial skeleton, and that different stimuli induce apparent variations in BMA, though the progenitor/BM adipocyte hierarchy and the relationship to skeletal stem cells remains to be illuminated.

The complete list of scientific sessions and contributors in each session is presented in Appendix A.

## Invited Speakers

Using diverse mouse models of marrow aplasia, *Bo Zhou* and *K Liu* focused on the role of BM adipocytes in hematopoietic regeneration. *Bo Zhou* showed that, in response to irradiation and BM transplantation, the cellular composition of the BM hematopoietic stem cell (HSC) niche changes since HSC maintenance and hematopoietic regeneration are promoted by BM adipocytes at different stages of commitment and BM stromal cells (BMSCs) (24). *K Liu* showed that adiponectin is one of the main molecules involved in the regulation of delayed hematopoietic recovery after chemotherapy or BM transplantation (36). *AJ van Wijnen* (23, 32) and *M Kassem* (21, 25) explored the molecular mechanisms involved in the lineage-specific commitment (adipocyte vs. osteoblast) of BMSCs, the understanding of which can provide significant insights into the process of age- and osteoporosis-related impaired osteogenesis. *MC Horowitz* and *DB Chou* presented data on novel technological tools to investigate BM adipogenesis. *MC Horowitz* presented details of a technique that couples histochemical staining of cell-bound lipids using osmium tetroxide with micro-Computerized Tomography (microCT) to visualize and quantify mouse BMA and underlined the importance of linear tracing experiments to assess the ontogeny of BM adipocytes (20)*. DB Chou* discussed the ongoing development of human BM on a chip, a microfluidic device able to reproduce important aspects of the *in vivo* microenvironment, detailing its performance in recapitulating radiation-induced acute myelosuppression. *F Pflumio* focused on the role of different BM sites in orchestrating human and mouse T-cell acute lymphoblastic leukemia to convincingly demonstrate that adipocyte-rich sites contribute to drug-resistant leukemic cells escaping treatment (19).

## Selected Speakers

A substantial part of the selected presentations used different mouse models to focus on the ontogeny of BMA; the relationship between hematopoiesis, bone tissue, and BMA; the endocrine and paracrine regulation of BM adipocytes; the role of BMA in diseases. *CS Craft*, using adiponectin-Cre^DTA+/−^ and Uncoupling-Protein-1(UCP1)-Cre^DTA+/−^, provided critical insights into the region-specific properties of BM adipocytes, particularly regarding the expression of adiponectin and the capacity of some BMAT depots to induce beiging. Data on Tyrosine Kinase Substrate with four SH3 domains (Tks4), inorganic Phosphate Transporter-2 (PiT2), and adiponectin receptor-1 (AR-1) deficient mice were the subjects of the presentations by *V Vas, S Beck-Cormier*, and *A Sowman*, respectively. Tks4, a scaffold protein involved in podosome formation, *Epithelial Growth Factor Receptor* (EGFR) signaling, and *Reactive Oxygen Species* (ROS) production, was shown to be necessary for both the adipogenic and osteogenic differentiation of BMSCs (37). The phenotypic characterization of PiT2 (reduced bone volume, impaired mineralization, altered biomechanical parameters, increased circulating alkaline phosphatase, and an increase in BMA in the absence of significant changes of the plasma levels of adiponectin) and AR-1 (low bone mass due to decreased osteoblast formation and increased BMA) knockout mice also supported a role for both molecules in the modulation of BMSC lineage commitment and differentiation toward adipocyte or osteogenic lineages. *TH Ambrosi* delineated the ontology of BM adipose tissue and the molecular identity of bone-resident adipocytes, establishing their involvement in age-dependent dysfunction of bone and hematopoietic regeneration (18). *A Wilson* explored hematopoiesis within the BM of lipodystrophic mouse models entirely lacking adipocytes, including the epiblast-specific PPAR-γ deletion, and reported that the lack of BM adipocytes alters the HSC niche and contributes to the onset of severe extramedullary hematopoiesis (34). Mattiucci et al. explored the role of BM adipocytes isolated from hip surgery patients in supporting HSC survival (22). He showed that short-term hematopoietic progenitors would not proliferate in the presence of primary human BM adipocytes as compared to undifferentiated BMSC controls, but HSCs survived and could form colonies after 5 weeks of co-culture with primary BM adipocytes, thus supporting the role of BM adipocytes in the hematopoietic niche. *E Douni* described two transgenic RANKL mouse lines wherein the number of copies of the transgene correlates with the severity of the osteoporotic phenotype, emphasizing their suitability as models for investigating the pathogenetic mechanisms that regulate the development and expansion of BMA in osteoporosis. *EL Scheller* focused on the regulation of BM adipose tissue by peripheral sympathetic nerves (29, 31); in this context, she identified regulatory pathways and sites within the brain with the potential to coordinate BMA in concert with peripheral adipose depots. Based on *in vitro* and *in vivo* data, *A Perino* showed that the bile acid-responsive membrane receptor TGR5 promotes mitochondrial fission through the ERK/DRP1 pathway and demonstrated beige remodeling of subcutaneous white adipose tissue under multiple environmental cues, including cold exposure and prolonged high-fat diet feeding (33).

Three of the selected presentations focused on technologies and engineering approaches for assessing BMAs. A novel microCT contrast agent for the three-dimensional simultaneous visualization of mineralized and soft skeletal tissue, the Hafnium-substituted polyoxotungstate, was presented by Kerckhofs et al. (30). Bourgine et al. (26) presented a three-dimensional *in vitro* biomimetic engineered human BM analog that exhibited compositional and structural features of human BM while supporting the maintenance of HSCs and progenitors. The metabolic functions of the BM adipose tissue in mice and humans, as revealed by [^18^F]-FDG-PET/CT imaging, were illustrated by *K Suchacki*.

Other selected presentations explored the relationship between BMA and diverse pathologic conditions. *N Al Rassy* showed that moderate physical activity does not prevent BMA accumulation in a mouse model of chronic food restriction. Using the separation-based anorexia (SBA) mouse model, *C Chauveau* suggested that corticosterone is involved in the BMA regulation of SBA mice and demonstrated the impact of the differentiation of BMSCs toward the adipocyte lineages as well as of the stimulated bone resorption on the reduced bone mass. Data on the potential role of the Methyl-CpG binding protein-2 (MeCP-2) and its related microRNAs in BMSC-derived adipogenesis were presented by *MR Rippo*. Finally, with her study on BMSCs isolated from BM aspirates obtained from lean, overweight, and obese men, *M Tencerova*, reported that BMSCs from obese men maintained insulin responsiveness and suggested that the enhanced adipogenic differentiation potential of BMSCs may lead to their exhaustion, contributing to the impaired bone formation and bone fragility observed in obesity.

## Posters

The seven posters covered different aspects of BMA, including the lack of involvement of the Peroxisome Proliferator-Activated Receptor-γ (PPAR-γ) pathway in the accumulation of BMA in an ovariectomy mouse model (*K Beekman*); the use of digital holographic microscopy for quantifying lipid content during adipocyte differentiation *in vitro* in a non-perturbing manner (*V Campos*; 25); the long-term effect of ovariectomy on mandibular bone microarchitecture and BMA in a comparison of the tibia of adult rats (28); the expression of brown fat genes during adipocytic differentiation and its modulation by glucorticoid in rat proximal femur-derived BMSCs (*WF Ferris*); the phenotypic characterization of mice with targeted expression of Gαs^R201C^ in mature adipocytes (*R Labella*); the molecular characterization of new potential markers of BM adipocytes (*G Maurizi*); and the association of increased BMA with chronic kidney disease (35).

## Creation of the International BMAS

The creation of a scientific society dedicated to BMA was envisioned as early as 2015 when a first group of physicians and scientists was formed (the BoneAHeaD group, BONE Adiposity in HEAlth and Diseases) with a shared need for an international society that focused on BMA. This group was funded by the French Research Agency with the goal of building a European network and applying to international funding calls. The need for the creation of a society was repeatedly posed during the 1st [BMA 2015, https://bma2015.sciencesconf.org: (3)] and 2nd BMA meetings [BMA 2016, https://adiposity.sciencesconf.org/; (4)] and in the last meeting of the BoneAHeaD group that took place in Boulogne-sur-Mer, France, in March 2017. At the BMA 2017, a preparatory meeting was organized on August 30th, 2017 (15:30–20:00) at EPFL in which members of the BoneAHead group invited speakers from the three BMA meetings (BMA 2015, BMA 2016, and BMA 2017) and other researchers who expressed an interest in the creation of an international society dedicated to BMA. The purpose of this preparatory meeting was to elaborate a consensus proposal regarding the name, aims, statutes, and general organization of the society that were to be presented and voted on at the constitutive assembly (CA) that was held during the BMA 2017 (September 1st, 2017, 12:15–13:00). All participants of BMA 2017 were invited to the CA and also constituted the general assembly (GA). The name and aims of the society and the criteria for the composition and missions of the GA, executive board (EB), and scientific board (SB) were proposed, voted on, and unanimously approved by the CA. The interim (founding) EB and SB, the procedures to elect the definitive EB and SB, and the locations of upcoming meetings were also proposed, voted on, and unanimously approved. For the composition of the definitive EB and SB, priority was given to an appropriate gender, geographical, and scientific background balance. The aims of the International BMAS are the organization of BMA meetings, the research and training of young researchers in the BMA field, data sharing, establishing common research standards, fostering collaborations, and public engagement. In the statutes of the BMAS, the founding executive and scientific boards reflect the importance of maintaining rigorous research and guidelines within the society and BMA community at large. Consequently, to work toward the missions of the society, three working groups were created (nomenclature, methodologies, and biobanking) and three (public engagement, data repositories, and sponsorship) planned and definitely established soon after. Finally, the legal status and organization of the society was proposed, voted on, and unanimously approved. Statutes of the society were documented in French and English following the meeting and signed by members of the first EB on February 2nd, 2018. In the meantime, a society website (http://bma-society.org/) has also been created. From its beginning on September 1st, 2017 in Lausanne, where the BMAS headquarters are located, the society seeks to advance the knowledge of BMA by facilitating interdisciplinary exchanges, developing research strategies, and promoting the emergence of new ideas and concepts eventually aimed to improve understanding and treatment of the numerous diseases in which BMA is involved.

## Concluding remarks and Perspective on the International BMAS

Over the past 5–10 years, new research has massively advanced our understanding of BMA development and functions. BMA has been recognized as an active tissue involved in diverse biological processes (i.e., skeletal remodeling, hematopoietic and metabolic regulation), clinical conditions (i.e., caloric restriction, anorexia nervosa, osteoporosis, leukemia, multiple myeloma, cancer induced bone disease), and in aging. However, many biological and physiopathological questions regarding BMA remain unaddressed. For example: Which is the true identity of BM Adipocyte progenitors? Is there only one BM Adipocyte progenitor? How do different BM adipocyte progenitor change upon skeletal maturation and aging? What is the role of BMA in BM homeostasis? How do BM Adipocytes metabolically interact with their neighboring cells? How do BM Adipocytes and their immediate progenitors contribute to regulation of hematopoiesis and the HSC niche? How is BMA involved in osteoblastogenesis and osteoclastogenesis? Which factors (i.e., metabolites and hormones) do BM Adipocytes produce? Can we develop tools to address clear cause-effect relationships between BMA and disease beyond the now abundant correlative studies? Can BM adipocytes serve as an energy source for HSCs and BMSCs? Why (and how) BMA volume changes do occur in different clinical conditions (i.e., diabetes, obesity, anorexia nervosa)? What is the role of BM Adipocytes in the context of whole-body energy balance? What is the role of BM Adipocytes in the development of BM metastasis?

BMA 2017 has been a success both for the fruitful discussions resulting from the scientific program and collaborative spirit of the attendees, and for the creation of an international scientific society, the International Bone Marrow Adiposity Society (BMAS). In the short term, BMAS has set itself the ambitious task of establishing a common, consensual nomenclature, of identifying the technical challenges specific to the study of BMA and of working toward the harmonization of methodologies and biobanking approaches to encourage data sharing and collaboration. The Authors' perspective is that the BMAS will hold the community together, ensuring its continuity, and will inevitably contribute to the in-depth biological and functional characterization of BMAT, also catching the interest of young physicians, scientists, engineers and the public at large.

## Author Contributions

All authors listed have made a substantial, direct and intellectual contribution to the work, and approved it for publication.

### Conflict of Interest Statement

The authors declare that the research was conducted in the absence of any commercial or financial relationships that could be construed as a potential conflict of interest.
