# Supplementary material for: Brief Report From the 3rd International Meeting on Bone Marrow Adiposity (BMA 2017)
Source: Front Endocrinol (Lausanne). 2019 May 28;10:336. doi: 10.3389/fendo.2019.00336 (PMC6546805; doi:10.3389/fendo.2019.00336)
Supplement: Supplementary file 1 [file Data_Sheet_1.docx]

**Appendix A: Scientific sessions and list of contributors**

***Session 1: Developmental origin and properties of bone marrow adipocytes***

*Bo O Zhou* (University of Chinese Academy of Sciences, Shanghai, China), *Clarissa Craft* (Washington University, Saint Louis, MO, USA), and *Virag Vas* (Hungarian Academy of Sciences, Budapest, Hungary).

***Session 2: Bone marrow adipocytes and hematopoiesis***

*Kaiyan Liu* (Peking University Institute of Hematology, Beijing, China), *Thomas Ambrosi* (German Institute of Human Nutrition, Nuthetal, Germany), *Anne Wilson* (University of Lausanne, Lausanne, Switzerland), and *Domenico Mattiucci* (Università Politecnica delle Marche, Ancona, Italy).

***Session 3: Endocrine and paracrine regulation of bone marrow adipocytes***

*Andre J van Wijnen* (Mayo Clinic, Rochester, MN, USA), *Erica L Scheller* (Washington University, Saint Louis, MO, USA), and *Cristophe Chauveau* (ULCO University, Boulogne-sur-Mer, France).

***Session 4: Bone marrow adipocytes and bone***

*Moustapha Kassem* (University Hospital of Odense, Odense, Denmark), *Eleni Douni* (University of Athens, Athens, Greece), and *Sarah Beck-Cormier* (INSERM, Nantes, France).

***Session 5: Technologies and engineering approaches for assessing bone marrow adiposity***

*Mark C Horowitz* (Yale University School of Medicine, New Haven, CT, USA), *David Benson Chou* (Wyss Institute for Biologically Inspired Engineering at Harvard University, Boston, MA, USA), *Greet Kerckhofs* (KU Leuven, Leuven, Belgium), *Karla Suchacki* (University of Edinburgh, Edinburgh, UK), *Thibaut Klein* (Universität Basel, Basel, Switzerland), *Aneka Sowman* (University of Oxford, Oxford, UK), and *Nathalie Al Rassy* (ULCO University, Boulogne-sur-Mer, France).

***Session 6: Bone marrow adiposity and diseases***

*Françoise Pflumio* (INSERM, Fontenay-aux-Roses, France), *Maria Rita Rippo* (Università Politecnica delle Marche, Ancona, Italy), *Alessia Perino* (EPFL, Lausanne, Switzerland), and *Michaela Tencerova* (University of Southern Denmark, Odense, Denmark).

***Poster session***

*Kerensa Beekman* (VU University Medical Center, Amsterdam, the Netherlands), *Vasco Campos* (EPFL, Lausanne, Switzerland), *Xavier Coutel* (University of Lille , Lille, France), *William F Ferris* (Stellenbosch University, Cape Town, Western Cape, South Africa), *Rossella Labella* (Sapienza University of Rome, Rome, Italy), *Giulia Maurizi* (Università Politecnica delle Marche, Ancona, Italy), and *Gina Woods* (University of California, San Diego, CA, USA).

***Debate on the origin of BM adipocytes***

*Mark C Horowitz*, *Moustapha Kassem* (moderator*), Tim Schulz* (German Institute of Human Nutrition, Nuthetal, Germany), and *Bo O Zhou*.
